# A systematic review of childhood obesity in the Middle East and North Africa (MENA) region: Prevalence and risk factors meta-analysis

**DOI:** 10.12715/apr.2017.4.8

**Published:** 2017-06-15

**Authors:** Nesrine S. Farrag, Lawrence J. Cheskin, Mohamed K. Farag

**Affiliations:** 1 Department of Public Health and Community Medicine, Mansoura University Faculty of Medicine, Mansoura, Egypt; 2 Department of Health, Behavior & Society, and Global Obesity Prevention Center, Johns Hopkins Bloomberg School of Public Health, Baltimore, MD, USA; 3 Epidemiology Department, Johns Hopkins Bloomberg School of Public Health, Baltimore, MD, USA

## Abstract

Obesity rates are rising globally, but there is evidence that young people in the Middle East and North Africa (MENA) region are at particularly high risk. We systematically searched the literature to map the MENA region for prevalence of childhood overweight and obesity, and examine the underlying risk factors and adverse effects associated with obesity in this region. Inclusion criteria were: English-language, non-basic-science focused articles that used any of the standard obesity definitions and were conducted in the MENA countries within the last five years. We searched PubMed using combinations of key terms ((childhood) OR adolescence) AND obesity) AND (MENA or each country) AND (“last five years” [PDat]). Studies demonstrated an increasing prevalence of obesity among many countries in the MENA region, especially in the Gulf area. Notably, in Kuwait, prevalence rates of overweight and obesity were 25.6% and 34.8% among young males and 20.8% and 20.5% among females. A meta-analysis revealed that physical inactivity, increased screen time, and higher social status were risk factors for childhood obesity. Childhood and adolescent obesity is a major challenge facing countries of the MENA region. Further research is needed to fully investigate the role of nutrition and other specific risk factors and evaluate various interventions to manage this pervasive and growing health problem.

## Introduction

Chronic non-communicable diseases (NCDs) represent a global public health challenge. NCDs are already the predominant source of morbidity and mortality in high-income countries and are on track to dominate the health care needs of populations in most low and middle-income countries, primarily because of changes in lifestyle factors [1]. Obesity is one of the most important risk factors of the majority of NCDs. A strong evidence base is being built on the association of childhood obesity and early onset adulthood cardiovascular diseases, metabolic syndrome, type 2 diabetes, cerebrovascular diseases, cancers, and others [2]. Moreover, childhood obesity was found to be associated with premature death [3].

Worldwide, the prevalence of childhood overweight and obesity combined has risen by 47.1% between 1980 and 2013 [4]. The World Health Organization (WHO) reported that the global prevalence of childhood obesity has increased from 31 million to 42 million children, and increased in Africa alone from 4 to 10 million children during the period from 1990 to 2013 [3]. Although the rise in the prevalence of obesity is global, there are distinct geographical variations. The combined prevalence of overweight and obesity ranges from 10% in northern Europe to 40% in southern Europe among children below the age of 10 [5]; in Latin America, 20–30% of the population under 19 years of age are obese or overweight [6].

Underlying obesogenic factors are crucial for the prevention of obesity. Genetic studies have associated particular genes with body mass index (BMI), adipose tissue distribution, lean body mass, metabolic rate, and even lifestyle factors such as eating habits and physical activity level [7–9]. But the recent rise in the prevalence of obesity highlights the key role of environmental risk factors in this problem [10]. Several different studies have investigated the association of environmental risk factors with obesity, such as the quality and quantity of food intake, level of physical activity, sleep hours, screen hours, socioeconomic standard, and parents’ obesity. Although there is a consensus on the association of physical activity with obesity, the other risk factors gave variable results in different studies [9–11].

**Figure 1:**
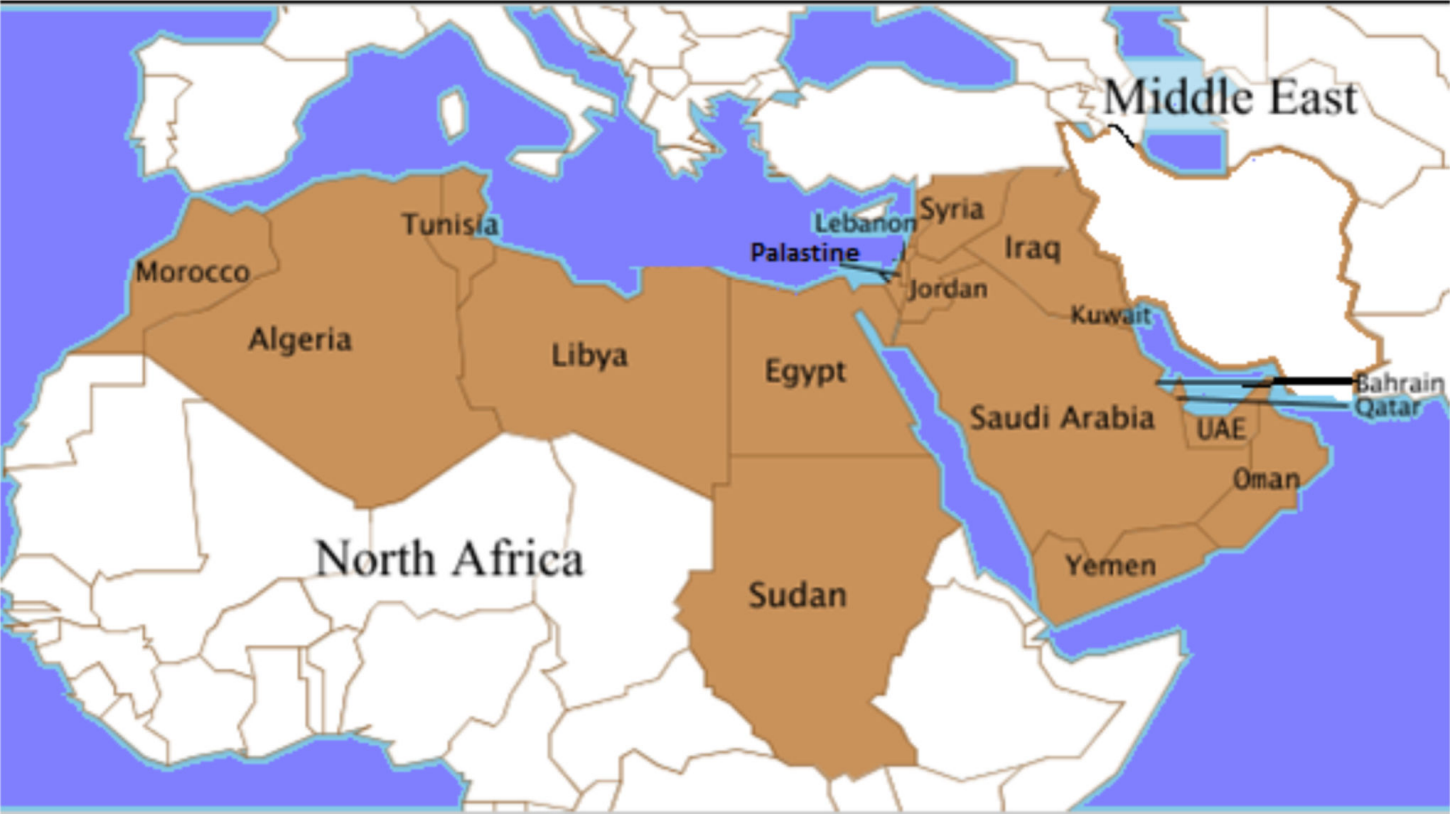
Map of the Middle East and North Africa (MENA) countries included in this study

The Middle East and North Africa (MENA) region covers an extensive geographic area that extends horizontally from Morocco to Iran, including 20 countries. However, there is some variance in the countries that are considered as being part of the MENA region; the WHO includes Sudan, Djibouti, and Iran, whereas the World Bank does not include Sudan [12, 13]. For the purpose of this review, 18 countries, constituting the vast majority of the MENA region and sharing a common language, culture, and traditions, are considered: Algeria, Bahrain, Egypt, Iraq, Jordan, Kuwait, Lebanon, Libya, Morocco, Palestine, Qatar, the Kingdom of Saudi Arabia (KSA), Sudan, Syria, Tunisia, the United Arab Emirates (UAE), and Yemen (Figure 1).

The population in this area is more than 300 million and most of the population lives in middle income countries. The MENA region – like many regions of the world – experienced a nutrition transition in the second half of the 20th century.

Urbanization and the adoption of a modern lifestyle together with the wealth of the oil-producing countries have contributed to the dramatic rise of obesity among all age groups and especially among children and adolescents in the region [12, 14].

Studies addressing the problem of overweight and obesity among children and adolescents in Arab countries are few or dated, and almost all used varying reference standards to measure obesity, making the comparison of obesity prevalence between countries difficult. Also, factors associated with the occurrence of obesity have not been well investigated in this region, which in turn adversely affects the ability to design effective programs to prevent obesity. National intervention programs to manage obesity in the Eastern Mediterranean are meager or absent [15]. One review published in 2010 reported the prevalence of obesity from 1990 to 2007 in most MENA countries but it did not address the risk factors of child obesity in these countries [16].

The objectives of our systematic review were to update the prevalence rates of overweight and obese children and adolescents in the MENA countries and underline the contributing risk factors. This paper represents the first part of a longer review; the second part includes the consequences of the problem of childhood obesity and management efforts in the MENA region.

## Methods

We performed a comprehensive systematic review of the latest studies – from early 2010 to August 2015 – reporting the prevalence of overweight and obese among male and female children and adolescents under the age of 20, regardless of color, race or religion.

Due to the scarcity of studies in many of the countries of the MENA region, we included studies that used any of the commonly-used standards in the diagnosis of overweight and obesity, including the International Obesity Task Force (IOTF), World Health Organization (WHO), and Centers for Disease Control and Prevention (CDC) standards [17]. One study in Qatar also used Qatar growth pattern curves.

Data were gathered from original research articles and systematic review articles that approached overweight and obesity in childhood and adolescence in the countries of the MENA region as a primary focus, or as a risk factor for other problems. We set the following inclusion criteria: non-basic science focused articles written in English that used any standard definition of obesity and were conducted in a MENA country within the last 5 years, whether reviews, observational, or interventional studies. We searched PubMed using combinations of key terms (((childhood) OR adolescence) AND obesity) AND (MENA) AND (“last 5 years” [PDat]) and the name of each country; for example (((childhood) OR adolescence) AND obesity) AND Kuwait AND (“last 5 years”[PDat]). The search strategy yielded 314 papers.

The data sets, titles, and abstracts were reviewed independently by two reviewers. We excluded 9 non-English language publications. After reviewing the titles, 161 articles were excluded due to unrelated topics. After examining the abstracts, 17 basic science studies, 53 papers that examined adults exclusively, 6 studies conducted outside the MENA region, one review of old studies, and 5 studies which included migrants from the MENA region residing in other countries were also excluded. After reviewing the full text of the remaining 62 papers, a further 6 studies were excluded: 3 miscellaneous studies, 2 that used a non-random sampling process, and 1 that was judged to have used inappropriate statistical analyses (frequent mistakes in the calculation of the significance level).

**Figure 2:**
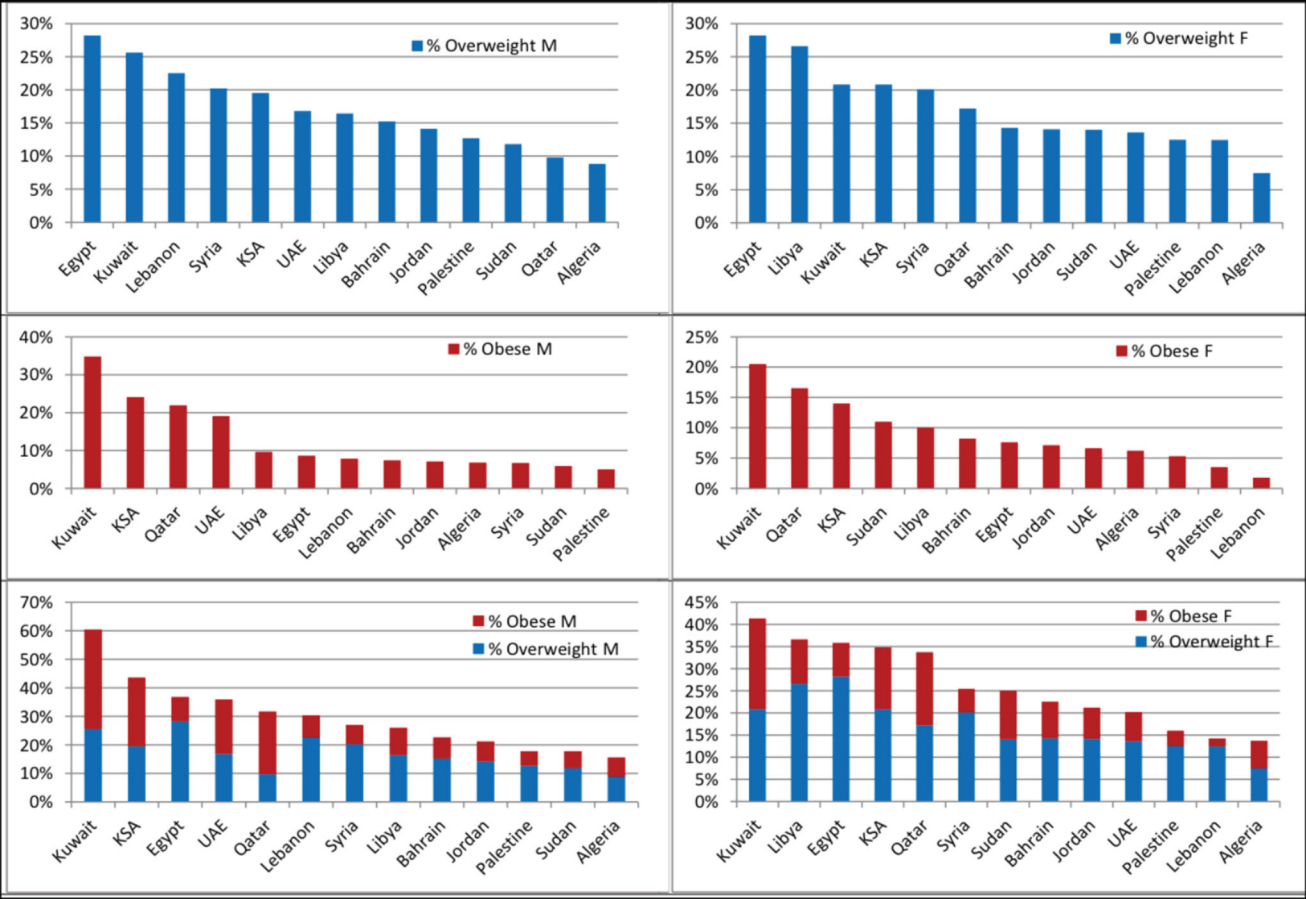
Ranking of Middle East and North Africa (MENA) countries by prevalence of overweight and obesity International Obesity Task Force (IOTF) standards are used among male and female children and adolescents; the rates reported are from original studies published in the last five years (since 2010). Five countries are not shown in this figure: Three countries which did not report original research studies in the last five years (Iraq, Oman, and Tunisia) and two countries which did not use the IOTF standards in BMI classification of their original research studies (Morocco and Yemen). BMI: Body Mass Index, IOTF: International Obesity Task Force.

We ranked the MENA countries by prevalence of overweight and obesity among children and adolescents based on the most recently reported data from original studies since 2010. To overcome the problem of different measurement standards, we only included studies that used the IOTF standards. We created separate tables for overweight and obese males and females, and for males and females using the sum of overweight and obesity (Figure 2).

A meta-analysis of predictive factors for childhood obesity was performed. The following potential predictors were analyzed: 1) physical activity, 2) gender, 3) diet, 4) screen time, 5) parental obesity, and 6) parental socioeconomic status (SES). Most of the studies used multivariable logistic regression to estimate the odds of childhood obesity as a function of various characteristics; some included generalized linear regression or a chi-squared test. We, therefore, selected log odds ratio as a measure of effect. For the studies that reported the beta coefficient from a linear regression model as the effect measure, we converted the results to a log odds ratio using methods described in Chinn [18].

STATA 14 (StataCorp. 2015) statistical software was used for the analyses [19]. The heterogeneity of the study-specific measures was assessed by means of the I2 statistic, which reflects the amount of between-study heterogeneity over and above the sampling variation, and is robust to the number of studies and choice of effect measure [20]. If the I2 statistic indicated high heterogeneity (greater than 50%), the summary measures were combined across the studies using the random-effect method, assuming that the included studies represented a sample from a larger population of studies [21]. To explore between-study heterogeneity, the effect of study-specific characteristics on the outcomes was estimated using meta-regression when the heterogeneity was high and the number of studies was above 5. We used the following potential predictors: children’s age, % male, mean BMI, and country.

## Results

Fifty-six articles met the inclusion criteria and were included in our review.

They included data on overweight and obese children and in Algeria [22–25], Bahrain [24], Egypt [26, 27], Jordan [28–33], Kuwait [34–40], Lebanon [41–45], Morocco [46], Palestine [47], Qatar [48–50], Saudi Arabia [51–63], Sudan [64], Syria [65], Tunisia [66, 67], UAE [68–72], Yemen [73, 74] and multi-country studies [4, 75, 76].

All studies were cross sectional, except for 3 reviews, 2 randomized controlled trials, 1 quasi-experimental study, and 1 retrospective cohort study.

### Prevalence

The specific prevalence rates obtained for each of the 18 MENA region countries are shown in Table 1. A recent systematic review estimated the 2013 prevalence of overweight and obesity in the MENA region in boys and girls less than 20 years old to be 22.2% and 27.9%, respectively [4]. However, the systematic review estimated the global, regional, and national prevalence of overweight and obesity from surveys, reports, and published studies that used only the International Obesity Task Force (IOTF) definition of childhood obesity [4].

It excluded several published studies that used other definitions, and excluded some sub-national studies. Nevertheless, the results of this study can be used for comparison of rates among different regions and countries but is less useful in providing age-related prevalence and trends since it lumped together the prevalence for all ages less than 20 years. Another useful study was conducted as part of a multi-center research project titled ARAB-EAT and included adolescents 15–18 years old from secondary schools in big cities in Kuwait, Libya, Palestine, Syria and UAE, using the IOTF definition of obesity. Only governmental schools were studied [76].

### Overall Rankings

The five countries with the highest prevalence of overweight children and adolescent males were: Egypt, Kuwait, Lebanon, Syria, and KSA (prevalence ranging from 28.2 to 19.5%). For females, the top five were: Egypt, Libya, Kuwait, KSA, and Syria (prevalence ranging from 28.2 to 20.1%). For obesity, Kuwait was by far the top country with a prevalence of 34.8 and 20.5% for male and female children and adolescents, respectively. When we ranked the MENA countries according to the sum of overweight and obesity children and adolescents (Figure 2), the top five countries for males were Kuwait (60.4%), KSA (43.6), Egypt (36.8%), UAE (35.9%), and Qatar (31.7%); whereas the top five countries for females were Kuwait (41.3%), Libya (36.6%), Egypt (35.8%), KSA (34.8%), and Qatar (33.7%).

### Risk factors

#### Physical activity

There is a general consensus that physical activity is protective against childhood obesity, but the results of studies in the MENA region are mixed. Al-Hazzaa et al. found that females in Saudi Arabia (Al-Khober, Jeddah, and Riyadh) were significantly more sedentary and less active than males (p < 0.001) [77]. Logistic regression analyses showed that higher physical activity was significantly associated with a higher consumption of fruits, vegetables, milk, French fries/potato chips and energy drinks [77]. However, in Amman, Jordan, Al-kloub et al. found that the level of physical activity was not associated with adolescent obesity [31]. The same result was found by Jildeh et al. in Palestinian adolescents [47].

On the other hand, Bener et al. found that less physical activity was a significant independent predictor of adolescent obesity in Qatar (OR=2.29, 95% CI= 1.45– 3.68) [49]. Mahfouz et al. found that lack of physical class exercise in the previous week (aOR = 1.452, 95% CI = 1.149–2.117) was associated with adolescents’ obesity in southwestern Saudi Arabia [53]. Additionally, Al-Hazzaa et al. showed that overweight/obesity (based on BMI categories) was significantly and inversely associated with vigorous physical activity levels (aOR for high level = 0.69, 95% CI 0.41–0.92 for BMI) in both males and females [59]. Al-Haif et al.’s study reported that among boys, moderate and vigorous activities w found to be significantly negatively associated with overweight and obesity (p < 0.001), whereas in girls, only those with not-less-than moderate activity levels were negatively associated with overweight and obesity (p < 0.001); the partial eta square explained by physical activity was less than 3.6% in boys compared with less than 1.0% in girls [36].

Overall, ten studies reported on the relationship between physical activity and childhood obesity; only seven of them reported the odds ratio of childhood obesity as a measure of association. Log-odds ratios of obesity (and their standard errors) associated with an increase in physical activity were calculated from all relevant studies.

**Figure 3:**
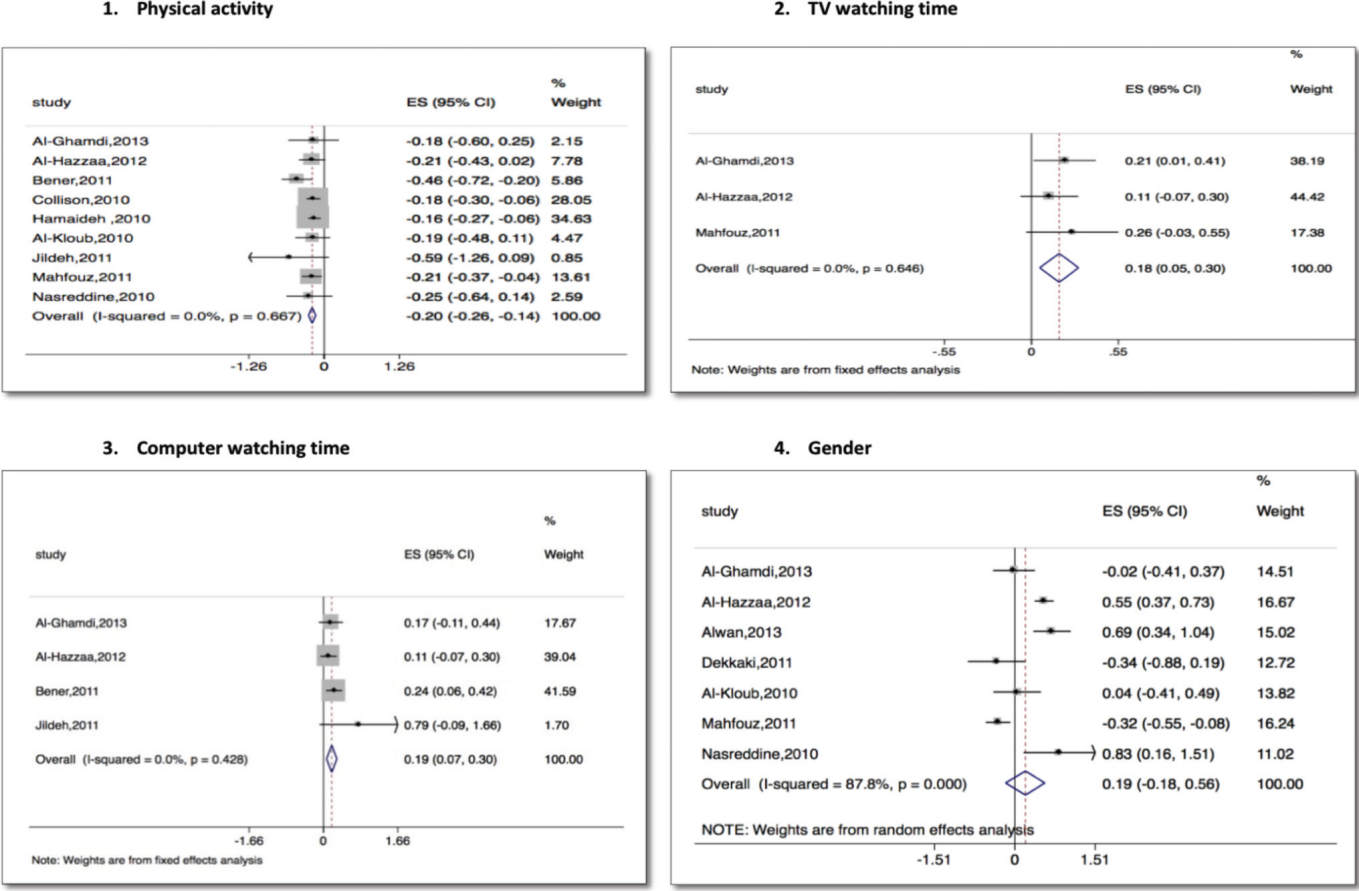
Meta-analysis of studies reporting risk factors of obesity (physical exercise, TV watching, computer watching, gender)

First, we analyzed only the 7 studies that reported odds ratios [31, 47, 49, 53, 59, 60, 65]. Next, we added two studies that reported beta coefficients [28, 52], dividing the log odds ratios (and their standard errors) by 1.81 to make them comparable to the reported beta coefficients [18]. The overall pooled effect of higher physical activity shows a log odds ratio of −0.46 (95% CI: −0.64 to −0.27) (Figure 3). The pooled result indicated no heterogeneity between the studies. After converting the log odds ratio into a normally-distributed measure and including 2 more studies, the overall pooled effect of higher physical activity is (beta coefficient) −0.20 (95% CI: −0.26 to −0.14).

#### Screen time

Al-Hazzaa et al. found that higher screen time (ST) was significantly associated with a higher consumption of sugar-sweetened drinks, fast foods, cake/doughnuts, and energy drinks [77].

Bener et al. found that excessive computer use was a significant independent predictor of adolescent obesity in Qatar (OR=1.27, 95% CI= 1.06–1.51) [49]. Al^−^ Ghamdi focused on the association between watching television and obesity in Saudi children aged 9–14, and found that the presence of only one television at home was associated with a 42% reduction in the risk of childhood obesity (OR = 0.58, p < 0.001). Moreover, the child’s personal ownership of a television was associated with an increased risk of obesity (OR = 1.75, p = 0.002). The study also found that as the number of hours of watching television on weekends decreased by one hour, there was an associated reduction of 19% in the risk of obesity (OR = 0.81, p = 0.009). However, personal computers and the Internet were not significantly associated with an increased risk of childhood obesity, as reported by Al-Ghamdi [60] and Al-Haif et al. found that time spent watching television and time spent working on the computer were not significantly associated with obesity in either sex [36].

**Figure 4:**
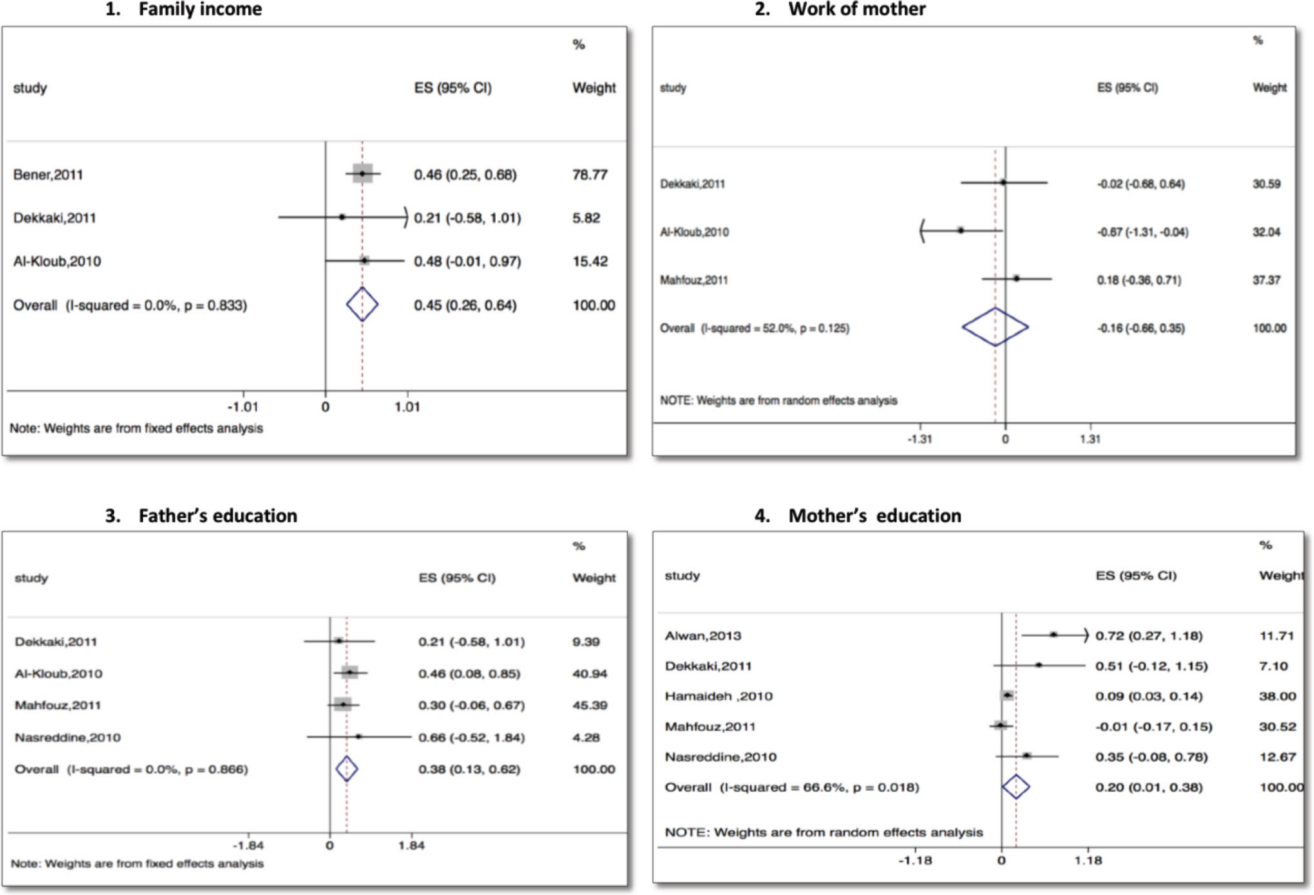
Meta-analysis of studies reporting risk factors of obesity (family income, work of the mother, father’s education, mother’s education)

Three studies reported odds ratios of childhood obesity in association with time spent watching TV (Figure 4) [53, 59, 60]. The standard error (SE) of the log odds ratio for this study was estimated by using the inverse of the standard normal distribution of the reported two-sided p-value. Increased TV watching was associated with increased odds of obesity – a pooled estimate of 1.19 (95% CI 1.06 to 1.35).

Four studies reported odds ratios of childhood obesity in association with time spent on the computer (Figure 4) [47, 49, 59, 60]. The SE of the log odds ratio for one study was estimated using the inverse of the standard normal distribution of the reported two-sided p-value. Computer time was associated with increased odds of childhood obesity – a pooled estimate of odds ratio 1.20 (95% CI 1.07 to 1.35).

#### Socio-economic standard

Al-Kloub et al. found that higher socioeconomic status (SES) in Amman, Jordan, put adolescents at higher risk of being overweight and obese. Using logistic regression, they found that family size ≤ 6 (OR = 1.59); income ≥ 300 Jordanian dinars (about $420/month) (OR = 1.62); working mothers (OR = 1.96); and father’s level of education ≥ secondary (OR = 1.59) were all significant predictors of excess weight [31].

In Saudi Arabia, the results of Alwan et al. were consistent with those from Jordan. They found by multivariate analysis that Saudi children were more likely to be overweight if they were male (female OR= 0.6, p < 0.01) 12 years of age (OR=3.79, p < 0.01, compared to age 6) and from a high-income family (OR=3.12, p < 0.01, compared to families with low income). They also reported that children were more likely to be obese if they were male (female OR=0.545, p < 0.01), aged 12 years (OR=3.9, p = 0.005, compared to age 6), and having a mother who was more educated. Mothers educated at the university level were found to have a three-fold higher risk of having obese children (OR=3.4, p < 0.01) compared to mothers with lower educational attainment [55]. However, Mahfouz et al. found that female Saudi adolescents were more likely to be obese (aOR = 1.372, 95% CI = 1.099–1.753) while all other sociodemographic variables (parental education, maternal occupation, urban/rural settings and consanguinity of parents) were non-significant [61].

In Qatar, Bener et al. reported that adolescent obesity was significantly associated with socioeconomic factors; namely, father’s education (p < 0.001), occupation (p < 0.001), family income (p < 0.0001), and number of bedrooms (p < 0.001), but not associated with mother’s education in bivariate analyses. When they used logistic regression, however, obesity was associated only with family income (p = 0.029) and number of bedrooms (p < 0.001) [49].

In Bahrain, Musaiger et al. conducted a study on adolescents aged 15–18 and found that the mother’s education was a risk factor for obesity among both males and females (p = 0.0167 and p = 0.007, respectively). Also, having a birth rank among siblings higher than 4 was a protective factor (p = 0.009) for boys only [24].

In Morocco, Dekkaki et al. found no significant relationship between parents’ education, occupation, or monthly income and obesity in children [46]. In Syria, Nasreddine et al. showed that the odds of obesity increased consistently but non-significantly with increasing educational attainment of both parents and with lower crowding index. They defined crowding index as the number of persons within the household divided by the number of rooms, excluding kitchen and bathrooms. A lower crowding index indicates a higher SES [65].

The meta-analysis results of three studies indicated a statistically significant positive association between higher family income and odds of childhood obesity – a pooled odds ratio of 1.57 (95% CI 1.30 to 1.91) (Figure 4) [31, 46, 49].

The analysis of four studies that reported odds ratios of childhood obesity related to mother’s education showed inconsistent results, with statistically significant increased odds of childhood obesity associated with higher mother’s education [46, 53, 55, 65]. The pooled odds ratio estimate from the random effects model was 1.88 (95% CI 0.94 to 3.76). The wide confidence interval indicates high uncertainty around the pooled estimate. In addition, we included one more study that reported a beta coefficient associated with mother’s education [28]. The pooled beta coefficient and its 95% CI estimated using random effects modeling is shown in Figure 4. Based on three studies, the relationship between childhood obesity and mother’s work outside home was inconclusive (Figure 4) [31, 46, 53].

The four studies of father’s educational attainment and odds of childhood obesity revealed a statistically significant positive association – a pooled odds ratio of 1.46 (95% CI 1.14 to 1.86) (Figure 4) [31, 46, 53, 65].

#### Diet

Although the hypothesis of a positive association between energy consumption and BMI is intuitively clear, the studies conducted in the MENA region were conflicting. For example, Nasreddine et al. found that energy intake from carbohydrates contributed significantly to the prevalence of obesity among Syrian adolescents (OR = 1.96, 95% CI: 1.06–3.16), even after controlling for total energy intake [65]. However, Jildeh et al. found that obese and overweight adolescents had lower energy intake (p < 0.05) than their normal-weight counterparts [47].

The Saudi Arabian study of Collison et al. showed a significant association between male BMI and sugar-sweetened carbonated beverage (SSCB) intake in a multivariate regression model (p < 0.0001). They also found that the intake of SSCB was positively associated with poor food choices like fast foods, savory snacks, and iced desserts, and total sugar consumption (p < 0.001) in both males and females. Consumption of SSCB and sugar-sweetened hot beverages were significantly higher in older versus younger children (p < 0.001) [52]. The same finding was detected by Nasreddine et al. among Syrian adolescents [42]. Another cross-sectional study, conducted in three cities in Saudi Arabia (Al-Khobar, Jeddah and Riyadh) found that overweight/obesity (based on BMI categories) was significantly and inversely associated with frequency of breakfast (aOR for < 3 days/week = 1.44; 95% CI 1.20–1.71 for BMI) and consumption of sugar-sweetened beverages (aOR for < 3 days/week = 1.32; 95% CI 1.08–1.62 for BMI) [59]. Additionally, the study by Al-Haif et al., which forms part of the Arab Teens Lifestyle Study (ATLS), showed that consumption of breakfast, vegetables, and fast foods (in Kuwaiti boys and girls) and potatoes, cakes and doughnuts, and sweets (in Kuwaiti girls only) was significantly associated with overweight and obesity (p < 0.05). In general, the partial eta square explained by eating habits was less than 1.8% in boys, compared with 2.5% in girls [36].

In contrast, the study by Mahfouz et al. found no significant value of food choices in predicting the development of obesity among adolescents in the southeastern region of KSA [53]. Similarly, Bener et al. found no association between consuming fast food and adolescents’ obesity in Qatari children [49]. Additionally, Al-Muammar et al. (2014) did not find any significant association between BMI of Saudi adolescent girls aged 12–15 and any of the dietary habits studied like the main meal (lunch), having breakfast, drinking water, eating fruits and vegetables, or eating while watching television [63]. A similar lack of association was reported by Al-kloub et al. (2010) among Jordanian adolescents [31]. Further clouding the complex picture of the effect of dietary intake on risk of obesity among Jordanian children is the work of Tayyem et al., who found that, while the prevalence of overweight and obesity was significantly higher among adolescents (14–18 years of age) in private schools than public schools, there was a significantly higher intake of sweets (p = 0.002) and French fries (p = 0.02) in public schools, and more frequent breakfast intake (p = 0.19) in private schools [32].

Results of meta-analysis of all studies that reported dietary risk factors indicated high heterogeneity and the absence of a pooled effect. For example: having a full breakfast (3 studies I2 = 92%) [28, 31, 59], snacking (2 studies, I2 = 65%) [31, 60], and low-quality food including fast food, sugar-sweetened beverages, fried food, and soft drinks (5 studies, I2 = 79%) [28, 31, 52, 53, 59]. Also, analysis of three studies found an insignificant relationship between regular fruit consumption and childhood obesity [31, 53, 59].

#### Other risk factors

Parental obesity was found to be associated with approximately a 3-fold increase in the odds of overweight (OR = 3.01; 95% CI: 1.61–5.63), and obesity (OR = 2.93; 95% CI: 1.09–7.86) among Lebanese adolescents [42]. In Amman, Jordan, Al-kloub et al. found that the presence of obesity in both parents was a significant independent predictor of adolescent overweight and obesity (OR = 2.16, 95% CI: 1.06, 4.42) [31]. Similarly, Nasreddine et al. showed that obesity among Syrian adolescents was significantly more prevalent among subjects reporting a positive family history of obesity (OR = 2.98 95% CI: 1.09, 8.11) [65]. On the other hand, Dekkaki et al. found that there was no significant relationship between parental BMI and obesity in Moroccan children [46]. Based on the results of the analysis of 4 studies, there was an inconclusive relationship between parental and childhood obesity (I2=56.5%) [28, 31, 46, 65].

Very few studies examined the association between sleep and obesity in the MENA region. Bener et al., using logistic regression analysis for the predictors of obesity among adolescents aged 11–18 in Qatar, showed that children who sleep ≤6 hours per day had significantly higher incidence of obesity (OR, 95% CI= 1.74 (1.26–2.55), p < 0.001) [49]. Similarly, Collison et al found that the BMI was negatively correlated with hours of sleep in both genders (p < 0.001) among Saudi children [52].

Seven studies were included in the analysis of the association between gender and obesity. Results showed significant heterogeneity across studies, and the pooled estimate from the random-effect meta-analysis shows no significant relationship (Figure 3) [31, 46, 53, 55, 59, 60, 65]. Meta-regression was used to identify if any of the sample characteristics explain the heterogeneity in study results. It suggests that differences in average BMI across studies might explain heterogeneity in odds ratio estimates (R-square = 37%).

## Conclusions

The MENA region has witnessed a striking and well-documented increase in rates of overweight and obese children and adolescents in the past generation. While data is not complete, the rate of increase appears to be faster than that experienced among adults in the region, and is particularly rapid in certain countries, especially in the Gulf area, particularly Kuwait, Qatar, and Saudi Arabia. The prevalence of childhood obesity is demonstrably higher in many countries than in Western countries, including the United States. Socioeconomic factors and changes in diet and lifestyle, as well as how children and adolescents spend their time vis-à-vis sedentary vs. active pursuits, appear to be major drivers of this rapid, recent increase in rates of overweight and obesity. The peak prevalence of obesity in males and females were reported in Kuwait at the ages of 15 and 16 years old (40% and 41% in males, and 37.3% and 18.4% in females) [76]. In Egypt, the combined prevalence of obesity and overweight among school adolescents aged 11 to 17 was 40.7%, with a higher prevalence of obesity among males (8.6%) than females (7.6%) [27].

According to the systematic review of Ng et al., Qatar and Kuwait had the highest prevalence of obesity of all the MENA countries among males (18.8%) and females (23.3%) <20 years old, respectively, while Tunisia and Yemen had the lowest rates of prevalence among girls (4.2%) and boys (1.7) under 20, respectively [4].

No original studies on the prevalence of childhood obesity in Tunisia and Yemen were published since 2010 and so these countries were not included in Figure 2, which ranked MENA countries according to recently reported prevalence rates. The difference between Kuwait and the countries with the next-highest male obesity prevalence is more than 10%: KSA (24.1%), Qatar (21.9%), and UAE (19.1%). Kuwait, followed by Qatar and KSA, showed the highest female prevalence of obesity (20.5, 16.5 and 14%, respectively). It is evident that obesity is very common in the oil-producing countries of the Gulf region. This rank was different from the rank of countries using rates reported by Ng et al.’s review [4]. Although both ranks showed that wealthy, oil-producing countries had the highest prevalence of obesity and overweight, there are some discrepancies. An example is Lebanon, which ranked the 3rd highest in prevalence of obesity among under-20 males according to Ng et al.’s study yet ranked 7th in our ranking of original studies [4].

Of note, there were marked differences in prevalence rates reported by different studies in the same country. For example, in Lebanon, one study that included school adolescents reported rates of obesity of 7.8% and 1.7% among males and females, respectively, using the IOTF standard [41]. However, another community-based study reported that 16.1% and 4.4% of males and female children (6–19 years) were obese using WHO growth charts [42]. But Ng et al.’s review gave a completely different rate of obesity (12.5%) among under-20 females [4]. Another example is the Qatar study by Bener et al., which gave much lower rates of obesity among males (5.6%) and females (4.8%) than other studies [49]. Possible explanations for these inconsistencies are variations in the age groups included in the studies and the use of different standards for measuring obesity. In Bahrain, one study reported that at the age of 10 years, the prevalence of obesity among boys using the WHO definition (13.7%) was double the prevalence than when using the IOTF definition (6.6%) [24]. Therefore, it is difficult to compare the results of studies between different countries in the region. The prevalence of obesity among boys was close to that of girls in most countries, but in some studies, there was a wide gender gap (with boys the more obese), most starkly in Saudi Arabia and Kuwait (Table 1).

The number of original studies on obesity among children and adolescents in MENA countries was generally low. Saudi Arabia had the highest number of studies addressing the problem and they covered many regions of the country (8 studies reporting prevalence rates; see Table 1). Other countries such as Iraq and Yemen had very few primary studies addressing obesity, possibly due to political and armed conflict in those countries. One study described the health profile of all Iraqi refugees who were screened in International Organization for Migration clinics in Jordan during June 2007–September 2009 before arrival in the United States. The study reported that using CDC growth charts, 14% of Iraqi refugees aged 2–19 years were overweight and 11% were obese [78].

A nutrition survey conducted in the Western Sahara camps in Algeria included (among others) children younger than 5 years old. The survey showed the double burden of malnutrition among Western Saharan refugees living in a protracted emergency: prevalence of global acute malnutrition (GAM) among these children was 9.1%, while 2.4% were overweight and 0.8% obese [23].

The number of studies that examined the risk factors of obesity in MENA countries was also low. All studies addressed physical activity as a risk factor of obesity and the meta-analysis concluded that higher physical activity is protective with an overall pooled effect of (beta coefficient) −0.20 (95% CI: −0.26 to −0.14). This result goes in line with another study that reported that physical activity is inversely associated with body fat percentage (β = −0.099, p = 0.027) [79]. Our conclusion supports the results of other systematic reviews that highlighted the role of physical activity in prevention of obesity among children [80, 81]. Physical activity has beneficial effects on fat distribution, blood pressure, cholesterol levels, and insulin resistance among obese and overweight children [82].

Diet was assessed as a risk factor of obesity in nearly all studies that addressed the problem. Although breast feeding is known to have a protective effect against child obesity, none of the studies studied its association with obesity [83]. Only 2 studies assessed the total energy intake, and they give conflicting results. Snack intake, breakfast intake, and vegetable and fruit intake were examined in 3, 5, and 5 studies, respectively. Low quality diet was examined differently; 2 studies examined sugar-sweetened beverages, 1 study examined fried food, and 1 study examined fast food. This variation was a problem in combining studies in the meta-analysis and affected the result of meta-analysis, which concluded that neither of the dietary items examined has any significant association with child obesity. This is not the case for the results of a systematic review which concluded that the intake of sugar-sweetened beverages is associated with obesity [84]. Also, Rosenheck stated that there is an unequivocal association between the consumption of fast food and obesity [85]. However, Brown found that the studies that examined school-based strategies of changing diet to reduce obesity among children gave inconsistent results [86].

As discussed before, obesity in MENA countries is more prevalent in wealthy oil producing countries. This may result in a conclusion that obesity is related to socioeconomic standard and especially to income. This conclusion is further confirmed by the results of our meta-analysis, which showed that higher family income and father’s education have a statistically significant positive association with the odds of childhood obesity. However, in European countries, childhood obesity was more prevalent in southern Europe and in population groups with lower income and lower education [5]. A cross-national survey study in China, Russia, and the United States gave different results. The study found that obesity was more prevalent among groups with high SES in China and Russia, but groups with lower SES were at higher risk of obesity in the US [87].

Only 2 studies assessed the sleep hours as a risk factor of obesity. Both of them found a significant negative association between the number of sleep hours and obesity. A recent systematic review that included only longitudinal studies also found that for every one hour increment in sleep duration per day the risk of obesity and overweight was reduced by 21% [88].

Our meta-analysis showed inconsistent associations between child obesity and parental obesity. On the other hand, a recent systematic review that included different countries and different study designs revealed a significant positive association between child and parents’ obesity but the strength of association varied with child age, weight status and their country’s economic level [89].

Childhood obesity is a significant public health problem and its consequences on healthcare systems are prone to increase. As such, setting strategies for the prevention and control of the problem should be a priority on the health agenda of MENA countries. More research is needed to document nationally comparable prevalence rates, to follow up the trends of the problem, to understand the environmental risk factors in every country and tailor the strategic plan according to these risk factors, and to test appropriate interventions for their effectiveness in the management of the problem.

Due to cost and time constraints we did not include gray literature, conference abstracts or articles from the Web of Science in this review. Also, we opted not to contact authors or scientists in the field of obesity. Due to significant culture and language differences we did not include Iran and Djibouti in this review despite them being counted among the MENA countries by UNICEF.

## Figures and Tables

**Table 1:** Prevalence of obese and overweight children and adolescents in MENA countries

Country	Study	Age (years)	Setting	Sample size	Diagnosis	Overweight			Obese		

						Overall (%)	M (%)	F (%)	Overall (%)	M (%)	F (%)
	Saker et al., 2011	6–8	Primary schools	1520	IOTF		8.8	7.5		6.8	6.2
Algeria	Grijalva-Eternod et al., 2012	0.5–<5	Household^a^	1608	WHO*	2.4			0.8		
	Ng et al., 2014	<20	Systematic review		IOTF		14	14.6		7.7	15.3
Bahrain	Musaiger et al., 2014	10–13	Schools	2146	IOTF		15.2	14.3		7.4	8.2
	Ng et al., 2014	<20	Systematic review		IOTF		13.1	16		9.3	10.7
	El-Metainy et al., 2011	2–16	Alexandria University Hospital	1465	IOTF	15.2			10.5		
Egypt	Manyanga et al., 2014	11–17	Schools	5179	WHO*	22.1	19.6	20.6	9.3	8.6	7.6
	Ng et al., 2014	<20	Systematic review		IOTF		18.8	25.1		12.7	14.4
Iraq	Ng et al., 2014	<20	Systematic review		IOTF		11.3	16.8		8.2	8.2
	Hamaideh et al., 2010	14–17	Schools	824	IOTF	19.1	17.2	21	6.3	5.7	7
	Khader et al., 2011 b	7–18	Household	1034	IOTF	13.7	11.3	15.5	10	12.4	8.2
Jordan	Al-Akour et al., 2012	13–18	Schools	1433	CDC**	17.6	18.9	16.9	7.8	9.5	6.2
	Al-Kloub et al., 2010	15–16	Schools	518	IOTF	17.5			9.6		
	Tayyem et al., 2014 c	14–18	Schools	735	IOTF	10.2			6.5		
	Ng et al., 2014	<20	Systematic review		IOTF		16.1	17.4		8	8
	Musaiger et al., 2013	15–18	Schools	628	IOTF		25.6	20.8		34.8	20.5
Kuwait	Zaghloul et al., 2013 d	1–18	Household	655	WHO*		22.7	22.4		25.3	17.5
	Abdelalim et al., 2012	10.3	Primary schools	1,213	CDC**		21.8			17.4	
	Ng et al., 2014	<20	Systematic review		IOTF		17.2	22.2		16.7	23.3
	Fazah et al., 2010	14–18	Schools	1000	IOTF		22.5	12.4		7.8	1.7
Lebanon	Nasreddine et al., 2014 e	6–19	Household	868	WHO*	30.8	37.9	23.7	10.3	16.1	4.4
	Ng et al., 2014	<20	Systematic review		IOTF		17.2	17.3		15.9	12.5
Libya	Musaiger et al., 2013	15–18	Secondary schools	540	IOTF		16.4	26.6		9.6	10
	Ng et al., 2014	<20	Systematic review		IOTF		18	19.6		14.5	22.1
Morocco	Dekkaki et al., 2011	7–14	Schools	1570	WHO*	5.1			3.6		
	Ng et al., 2014	<20	Systematic review		IOTF		14.6	17.8		7.9	9.1
Oman	Ng et al., 2014	<20	Systematic review		IOTF		16.1	26.9		8.4	15.4
	Jildeh et al., 2011	11–16	Schools f	313	IOTF	24.3	22	26	9.9	8.2	11.7
Palestine	Musaiger et al., 2013	15–18	Schools g	477	IOTF		12.7	12.5		5	3.5
	Ng et al., 2014	<20	Systematic review		IOTF		16	18.1		11.9	12.5
	Rizk and Yousef, 2012	6–11	Schools	315	IOTF		9.7	17.2		12.9	16.5
Qatar	Bener et al., 2011	6–18	Schools	2467	***		2.3	17.5		5.6	4.8
	Ng et al., 2014	<20	Systematic review		IOTF		15.7	6.6		18.8	15.5
	El Mouzan et al., 2010	5–18	Household	19317	WHO*	23.1	22.4	23.8	9.3	10.1	8.4
	Collison et al, 2010	10–19	Schools	9433	CDC**	15.5	14.3	16.7	21.1	25.8	15.7
	Mahfouz et al., 2011	11–19	Schools	1869	CDC**		11.5	15.5		11.8	13.9
	Al-Nakeeb et al., 2012	15–17	Secondary schools	1138	IOTF	18.2	16.9	19.5	18.3	19.5	17.1
KSA	Alwan et al., 2013	6–16	Schools	1243	WHO*	21.1	21.5	21.3	12.7	17.4	9.3
	Al-Hazzaa et al., 2013	14–18	Secondary schools	1648	IOTF	20.7	19.3	21.9	17.6	22.6	12.9
	Al-Hazzaa et al., 2014	14–19	Secondary schools	2908	IOTF		19.5	20.8	24.1	14	
	Ng et al., 2014	<20	Systematic review		IOTF		14.1	22.6		9.4	14.8
	Alenazi et al., 2015	16.7	Schools	523	CDC**		17.2			30.4	
Sudan	Salman et al., 2010	6–12	Schools	304	IOTF		11.8	14		5.9	11
	Ng et al., 2014	<20	Systematic review		IOTF		5.5	8.6		5.7	5.8
	Nasreddine et al., 2010	15–18	Schools	776	WHO*	18.9			8.6	11.7	5.6
Syria	Musaiger et al., 2013	15–18	Schools	1062	IOTF		20.2	20.1		6.7	5.3
	Ng et al., 2014	<20	Systematic review		IOTF		19	17.9		13.9	15.4
Tunisia	Ng et al., 2014	<20	Systematic review		IOTF		13.5	19.2		4.2	4.2
	Musaiger et al., 2013	15–18	Schools	485	IOTF		16.8	13.6		19.1	6.6
	Al Junaibi et al., 2013	6–19	Schools	1440	CDC**	14.7	11.7	17.6	18.9	17	20.7
UAE		6–10		253			9.1	23.6		15.9	17.1
	Ng et al., 2012	11–18	Household	276	IOTF		16.2	20.5		11.7	19.7
	Ng et al., 2014	<20	Systematic review		IOTF		18.6	19		12.1	12.6
Yemen	Badi et al., 2012	6–16	Schools	1885	WHO*	12.7			8		
	Ng et al., 2014	<20	Systematic review		IOTF		7.4	18.6		17	8.3

WHO*: WHO reference (2007);

CDC**: CDC growth charts;

*** Qatari growth pattern curves were used

(a) in Western Sub-Sahara;

(b) the rates reported in the table were for adolescents:

(c) the reported rates are for public schools (the rates in private schools are higher);

(d) the reported rates are for ages 14–18;

(e) the reported rates are for ages 12–19;

(f) schools in East Jerusalem, Palestine;

(g) schools in Al-Khalil, Palestine
